# Proteomics, Transcriptomics, and Phosphoproteomics Reveal the Mechanism of Talaroconvolutin-A Suppressing Bladder Cancer *via* Blocking Cell Cycle and Triggering Ferroptosis

**DOI:** 10.1016/j.mcpro.2023.100672

**Published:** 2023-10-21

**Authors:** Yong Xia, Longquan Xiang, Ming Yao, Zhiying Ai, Wei Yang, Jianhua Guo, Shuhao Fan, Ning Liu, Xiaolong Yang

**Affiliations:** 1 Precision Medicine Laboratory for Chronic Non-communicable Diseases of Shandong Province, Institute of Precision Medicine, Jining Medical University, Jining, Shandong, China; 2 Department of Pathology, Jining No. 1 People's Hospital, Jining, Shandong, China; 3 School of Pharmaceutical Sciences, South-Central Minzu University, Wuhan, China; 4 College of Basic Medicine, Jining Medical University, Jining, Shandong, China

**Keywords:** bladder cancer, proteomics, transcriptomics, phosphoproteomics, pharmacology

## Abstract

Talaroconvolutin-A (TalaA) is a compound from the endophytic fungus *T. convolutispora* of the Chinese herbal medicine Panax notoginseng. Whether TalaA exerts anticancer activity in bladder cancer remains unknown. Using CCK8 assay, EdU staining, crystal violet staining, flow cytometry, living/dead cell staining, and Western blotting, we studied the anticancer activity of TalaA *in vitro*. Moreover, we performed xenograft tumor implantation. The antitumor effects were evaluated through H&E and immunohistochemistry staining. Proteomics was conducted to detect changes in the protein profile; transcriptomics was performed to detect changes in mRNA abundance; phosphoproteomics was used to detect changes in protein phosphorylation. TalaA inhibited tumor cell proliferation, DNA replication, and colony formation in a dose-dependent manner in bladder cancer cells. The IC_50_ values of TalaA on SW780 and UM-UC-3 cells were 5.7 and 8.2 μM, respectively. TalaA (6.0 mg/kg) significantly repressed the growth of xenografted tumors and did not affect the body weight nor cause obvious hepatorenal toxicity. TalaA arrested the cell cycle by downregulating cyclinA2, cyclinB1, and AURKB and upregulating p21/CIP. TalaA also elevated intracellular reactive oxygen species and upregulated transferrin and heme oxygenase 1 to induce ferroptosis. Moreover, TalaA was able to bind to MAPKs (MAPK1, MAPK8, and MAPK14) to inhibit the phosphorylation of ∗SP∗ motif of transcription regulators. This study revealed that TalaA inhibited bladder cancer by arresting cell cycle to suppress proliferation and triggering ferroptosis to cause cell death. Conclusively, TalaA would be a potential candidate for treating bladder cancer by targeting MAPKs, suppressing the cell cycle, and inducing ferroptosis.

## Highlights

• To study the anticancer effect of Talaroconvolutin-A against bladder cancer *in vivo* and *in vitro*.
• Use of proteomics, transcriptomics, phosphoproteomics to explore the anticancer mechanism.
• TalaA can block the cell cycle and induce ferroptosis.
• TalaA suppresses the phosphorylation of ∗SP∗motif by interacting with MAPKs.

## Graphical Abstract

TalaA inhibits bladder cancer mainly through: (1) arresting cell cycle to suppress proliferation, (2) triggering ferroptosis to cause cell death. In molecular mechanism, TalaA inhibits the protein kinase activity through interaction with MAPKs (including but not limited to MAPK1, MAPK8, and MAPK14), resulting in a decrease of the phosphorylation level of downstream substrates, including important transcription factors of cancer cells (JUN and NFATC1), translation regulatory elements (EIF4EBP1 and EIF4ENIF1), and important signal transduction molecules of cancer cells (ABL1 and RPS6KB1). The decrease of the phosphorylation level of the above proteins will reduce the transcription efficiency and protein abundance of cell cycle–related proteins such as AURKB, CCNB1, CCNA2, and other molecules, so as to repress cell cycle and inhibit proliferation. MAPK, mitogen-activated protein kinase; RPS6KB1, ribosomal protein S6 kinase B1; TalaA, talaroconvolutin-A.

**Figure:**
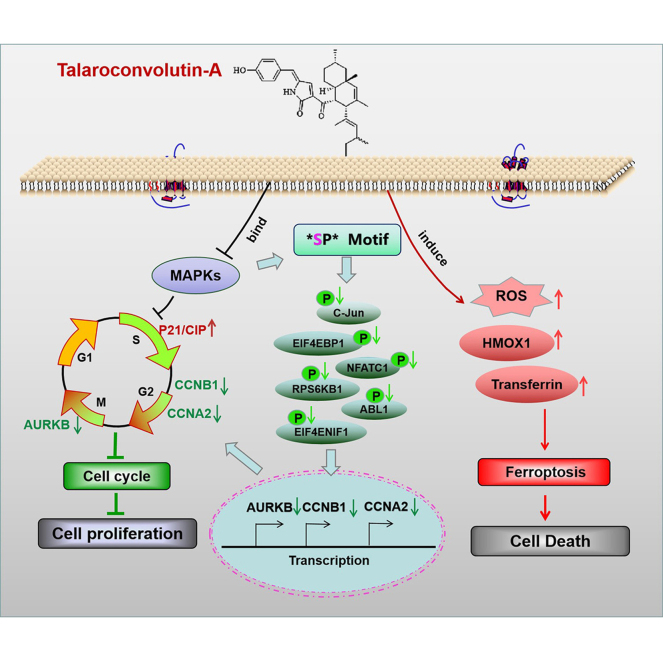

## In Brief

Existing chemotherapeutic drugs for bladder cancer are limit. Talaroconvolutin-A (TalaA) is a novel bioactive constituent extracted from the endophytic fungus *T. convolutispora* of the Chinese herbal medicine *Panax notoginseng*. This study reveals that TalaA inhibits bladder cancer mainly by arresting the cell cycle to suppress proliferation and triggering ferroptosis to cause cell death. TalaA interacts with MAPKs to repress the phosphorylation of transcription regulators. TalaA could be a potential candidate for treating bladder cancer.

Bladder cancer, the fourth most common cancer, is a malignant carcinoma that predominately affects the male population worldwide (1). Bladder cancer can be classified into nonmuscle invasive and muscle-invasive bladder cancers (2), both of which show high recurrence rates and result in great physical pain and economic burden to patients. Endoscopic resection and adjuvant therapy are often used to treat nonmuscle invasive bladder cancer, but the recurrence rate is high, necessitating chemotherapy to improve outcomes (3). Muscle-invasive bladder cancer exhibits a high rate of metastasis, which is the primary cause of death in these patients (4). For muscle-invasive bladder cancer and bladder cancer with distant metastasis, more radical treatment methods, including radical cystectomy and urinary diversion and triple therapy, involving resection, radiotherapy, and sensitization chemotherapy, are typically adopted to control the risk of metastasis and disease-specific mortality. Chemotherapeutic drugs, such as cisplatin, gemcitabine, doxorubicin, and epirubicin, cause DNA damage, prevent DNA replication, or induce cell apoptosis (5). However, these drugs cause toxic side effects, and drug resistance may develop; therefore, high-efficiency and low-toxicity candidate chemotherapy drugs are urgently needed.

Compounds derived from natural products have received extensive attention, and some have been developed as first-line anticancer drugs, for example, paclitaxel, camptothecin, and vincristine. However, the therapeutic effects of these drugs on bladder cancer require further analysis (6, 7). Therefore, screening and research of natural compounds with potential for treating bladder cancer are needed. The secondary metabolites of endophytic fungi of medicinal plants reportedly have anticancer activities (8, 9). Talaroconvolutin-A (TalaA), a secondary metabolite of the endophytic fungus *T. convolutispora*, which is used as a traditional Chinese herbal medicine, has been explored for its bioactive function. We previously reported the anticolorectal cancer activity of TalaA and the associated mechanism. TalaA increased reactive oxygen species (ROS) in colorectal cancer cells and affected the expression of proteins regulating lipid peroxidation, disrupting the oxidation-reduction balance and inducing ferroptosis (10). In this study, we found TalaA strongly inhibits bladder cancer cells, but the mechanism differs from that in colorectal cancer. Therefore, we aimed to verify the anticancer effect of TalaA against bladder cancer *in vitro* and *in vivo* and determine its pharmacological and molecular mechanisms at the transcription, protein, and phosphorylation levels. CCK8, 5-Ethynyl-2′-deoxyuridine (EdU) staining, clonogenesis, xenograft, and flow cytometry experiments demonstrated that TalaA strongly inhibited bladder tumors. Using integrated omics methods, including transcriptomics, proteomics, and phosphoproteomics, we revealed the comprehensive molecular mechanisms of the TalaA network pharmacology.

## Experimental Procedures

### Cell Culture and Main Reagent

SW780 and UM-UC-3 bladder cancer cells were used as research models for *in vitro* analysis. Cell lines were obtained from the BeNa Culture Collection. The cells were cultured in high-glucose Dulbecco’s Modified Eagle Medium (DMEM; HyClone) supplemented with 10% fetal bovine serum (FBS; HyClone) and antibiotics (100 μg/ml streptomycin and 100 U/ml penicillin). TalaA was extracted from endophytic fungus *T. convolutispora* in *Panax notoginseng* and purified as previously described (10). N-Acetyl-L-cysteine was purchased from Merck; deferiprone (DFO) and ferrostatin-1 were obtained from MedChemExpress (Monmouth Junction). Other chemicals are purchased from local reagent companies.

### Cell Activity Measurement

Cell proliferation and cell activities were measured using a commercially available Cell Counting Kit (CCK8, Dojindo) according to the manufacturer’s instructions. The reagent was added to each well, diluted (1:1000) in DMEM, and incubated for 30 to 40 min. Incubation was stopped when the absorbance value at 450 nm of the positive control (without anticancer chemicals) exceeded 0.7. Absorbance values were recorded using a microplate reader (Ensight Microplate Reader, PerkinElmer) at a wavelength of 450 nm.

### DNA Synthesis Measurement by EdU Staining

EdU is a thymidine analog that can be inserted into newly replicated DNA during DNA synthesis. We employed red fluorescent dye-labeled EdU (C0078L, Beyotime) to detect the inhibitory effect of TalaA on DNA synthesis in bladder cancer cells according to the manufacturer’s instructions. The EdU staining results were photographed using an inverted fluorescence microscope (Nikon).

### Colony Formation Assay

The colony-forming ability of cells reflects their proliferation ability. We conducted colony formation assays to study the long-term effect of TalaA on inhibition of the colony-forming ability of bladder cancer cells. The experiments were performed in a 12-well plastic dish. The initial number of cultured cells was 800 cells/well. After the cells had adhered to the walls of the plate, they were treated with different concentrations of TalaA. The culture media were changed every 3 days, and TalaA was also renewed. After culture for 12 days, the cells were stained with crystal violet, photographed, and imaged using a digital camera.

### Xenografts in Nude Mice

SW780 bladder cancer cells (10^7^ cells) were subcutaneously implanted into 12 female 6-week-old BALB/C-nude mice (Mice were obtained from Jinan Pengyue Laboratory Animal Breeding Co, Ltd). When the transplanted tumors grew to 60 mm^3^, the mice were randomly divided into two groups (six mice per group). The TalaA-treated group was intraperitoneally injected with 6.0 mg/kg TalaA (dissolved in dimethyl sulfoxide (DMSO) and then in corn oil), whereas the control group was administered the same volume of DMSO and corn oil. TalaA (or DMSO) was injected twice daily. The body weight of mice and tumor volume were recorded every 2 days. After 12 injection doses, the mice were euthanized by exposure to CO_2_, and the transplanted tumors were photographed and weighed. After fixation in 10% formalin for 24 h, the tumors, liver, and kidneys were dehydrated and embedded in paraffin blocks. Animal experiments were approved by the Experimental Animal Ethics Committee of Jining Medical University (Approval No. JNMC-2021-DW-003).

### Pathological Staining in Mice

The formalin-fixed samples were dehydrated in an automatic dehydrator (Yaguang Technology) and cut into 4 μm-thick slides using an automatic slicer (Leica). After heating at 60 °C for 1 h, the slides were subjected to gradient dehydration, followed by H&E and immunohistochemistry (IHC) staining. The H&E staining kit was purchased from Solarbio. Before staining, the samples on the slides were repaired with citric acid buffer, and endogenous hydrogen peroxide was inactivated with 3% hydrogen peroxide. After blocking with goat serum, the primary antibody (anti-Ki67) was co-incubated with the sample at 4 °C overnight (10–14 h). Rabbit anti-Ki67 (1:300, abcam 15580, Abcam) was used as primary antibody for IHC staining. After four washes in PBS, the slides were co-incubated with a polyclonal secondary antibody (SV0004, Boster) and diaminobenzidine developing solution. Positively stained cells were brown.

### Western Blotting

Western blotting was performed to quantitatively analyze whether TalaA altered protein levels in bladder cancer cells. The primary antibodies used in this study were rabbit anti-cycle checkpoint protein p21/CIP and reducing cyclin A (CCNA2; 1:1000; Proteintech, 18202-1-AP), rabbit anti-cyclin B1 (CCNB1; 1:1000, Proteintech, 28603-1-AP), rabbit anti-p21/WIF (1:1000, Cell Signaling Technology, #2947), mouse anti-GAPDH (1:1500, OriGene, TA802519), mouse anti-MAPK1 (1:2000, Cell Signaling Technology, #4696), rabbit anti-MAPK14 (1:1000, Cell Signaling Technology, #9212), and rabbit anti-β-actin (1:2000, Servicebio, GB111364). The secondary antibody (horseradish peroxidase–conjugated) (1:5000) was purchased from ABclonal, and the enhanced chemiluminescence detection solution was obtained from Beyotime.

### Proteomics Analysis

To explore the molecular mechanism of TalaA-mediated inhibition of bladder cancer, we performed proteomic detection experiments. The samples were divided into two groups: the TalaA treatment group (8 μM TalaA treated for 24 h) and the control group (equal volume of DMSO). Each group had three independent samples. SDT buffer (4% sodium dodecyl sulfate, 100 mM Tris–HCl, 1 mM DTT, pH 7.6) was used for sample lysis and protein extraction. The extracted protein was temporarily stored at −80 °C and transported on dry ice to Applied Protein Technology for enzymatic hydrolysis, labeling, and mass spectrometry (MS). Trypsin was used to generate the peptides. The number of permitted max missed and nonspecific cleavages was 2. The database information used to check the database is Swissprot_human_20368_20200217. The search parameters were set as follows: fixed modifications: carbamidomethyl (C), TMT6 plex (N-term), TMT6 plex (K); variable modifications: oxidation (M); mass tolerance for precursor ions: ±20 ppm, mass tolerance for fragment ions: 0.1 Da. False discovery rates of peptides and protein ≤0.01. Through searching the database, 6716 protein entries were identified. To identify and quantify the proteins, the MS raw data of each sample were searched using the MASCOT engine (version 2.2; Matrix Science) embedded in Proteome Discoverer 1.4. For screening of significantly different proteins, the threshold was set as follows, the fold change >1.2 (or <0.83) and *p* value <0.05. To predict protein subcellular localization, CELLO (http://cello.life.nctu.edu.tw/), a multiclass support vector machine classification system, was employed. Blast2GO software (https://www.blast2go.com/) was used for Gene Ontology (GO) enrichment mapping, and the GO enrichment map was plotted using R scripts. Proteome data were blasted against the Kyoto Encyclopedia of Genes and Genomes (KEGG) database.

### Combined Transcriptomics/Proteomics Analysis

The transcriptome, the main approach used to study alterations in gene expression, is the link between the genome (genetic information) and proteome (biological function). Transcriptome analysis was mainly focused on changes in mRNA abundance in bladder cancer cells (TalaA treatment group *versus* control group). Total RNA from the transcriptome was extracted using TRIzol (Invitrogen). The mRNA was purified using an mRNA purification kit (61006, Thermo Fisher Scientific). Library construction, sequencing, and bioinformatics analyses were performed by Applied Protein Technology. To comprehensively evaluate the effect of TalaA on the expression profile in bladder cancer cells, we integrated the transcriptome and proteome data at the gene and protein levels.

### Phosphoproteomics Analysis

Phosphorylation and dephosphorylation are important steps for controlling signaling pathways. To investigate the effect of TalaA on key signaling pathways in bladder cancer, we analyzed alterations in phosphorylation in TalaA-treated bladder cancer cells using phosphoproteomics analysis. Because the protein phosphorylation reaction is generally short and varies in length, we treated the bladder cancer cells with TalaA for 10 and 30 min. Therefore, the samples were divided into three groups: the TalaA shorter-time treatment group (10 min), TalaA longer-time treatment group (30 min), and the control group (DMSO). Each group had three independent samples. SDT buffer containing a 1% phosphatase inhibitor cocktail (MedChemExpress) was used for sample lysis and protein extraction. Phosphopeptides were enriched using a High-Select Fe-NTA Phosphopeptides Enrichment Kit (Thermo Fisher Scientific), and the peptides were determined by 10-label tandem mass tag (TMT) MS at Applied Protein Technology. The search parameters were set as follows: fixed modifications: carbamidomethyl (C), TMT10 plex (N-term), TMT10 plex (K); variable modifications: oxidation (M), phospho (ST), phospho(Y); mass tolerance for precursor ions: ±20 ppm, mass tolerance for fragment ions: 0.1 Da. MS/MS spectra were searched using the MASCOT engine (version 2.2; Matrix Science) embedded in Proteome Discoverer 2.4. Motifs were analyzed using MeMe (http://meme-suite.org/index.htm). For screening of significantly different phosphorylated proteins, the threshold was set as follows, the fold change >1.2 (or <0.83) and *p* value <0.05.

### ROS Detection

We used H_2_DCFDA (a ROS probe) to detect the effect of TalaA on intracellular ROS levels in bladder cancer. The medium of bladder cancer cells co-incubated with different concentrations of TalaA was replaced with a serum-free medium containing 2 μM H_2_DCFDA. After culturing in a 37 °C incubator in the dark for 30 min, the cells were digested with trypsin and analyzed using flow cytometry (Beckman Coulter).

### Mitochondrial Membrane Potential Analysis

To determine the effect of TalaA on the mitochondria of bladder cancer cells, a Mito Tracker Red cmxros Kit (c1049b; Beyotime) was used. After co-incubating the cells with different concentrations of TalaA, a medium containing 100 nm Mito tracker red cmxros without serum was added, and the cells were incubated at 37 °C for 30 min. The MiTo tracker red cmxros working solution was removed and replaced with fresh DMEM containing FBS. The cells were observed, and photos were acquired under a fluorescence microscope.

### Conjugation Biotin with TalaA

EDC·HCl (20 mg, 0.10 mmol), biotin (12 mg, 0.047 mmol), HOBt (11 mg, 0.082 mmol), and Et3N (16 mg, 0.16 mmol) were dissolved in anhydrous DMF (1.5 ml) under a nitrogen atmosphere. After 10 min, Tala A (19 mg, 0.039 mmol) was added and the reaction mixture was stirred at room temperature for 24 h. Then 5 ml water was added into the mixture and extracted with ethyl acetate. The combined organic solvent was washed with saturated brine, dried over anhydrous Na_2_SO4, and concentrated *in vacuo*. The Biotin-TalaA was purified by column chromatography (CH2Cl2/MeOH = 12:1).

### Statistical Rationale

Data from at least three experiments were statistically evaluated in each group. Significant differences between two groups were analyzed using Student's *t* test. One-way ANOVA was performed to compare multiple (more than two) groups, and LSD post hoc analysis is used to make pairwise comparisons. Differences were considered significant at *p* < 0.05.

## Results

### TalaA Inhibited Bladder Cancer Cell Growth *in Vitro* in a Dose-Dependent Manner

**Fig. 1:**
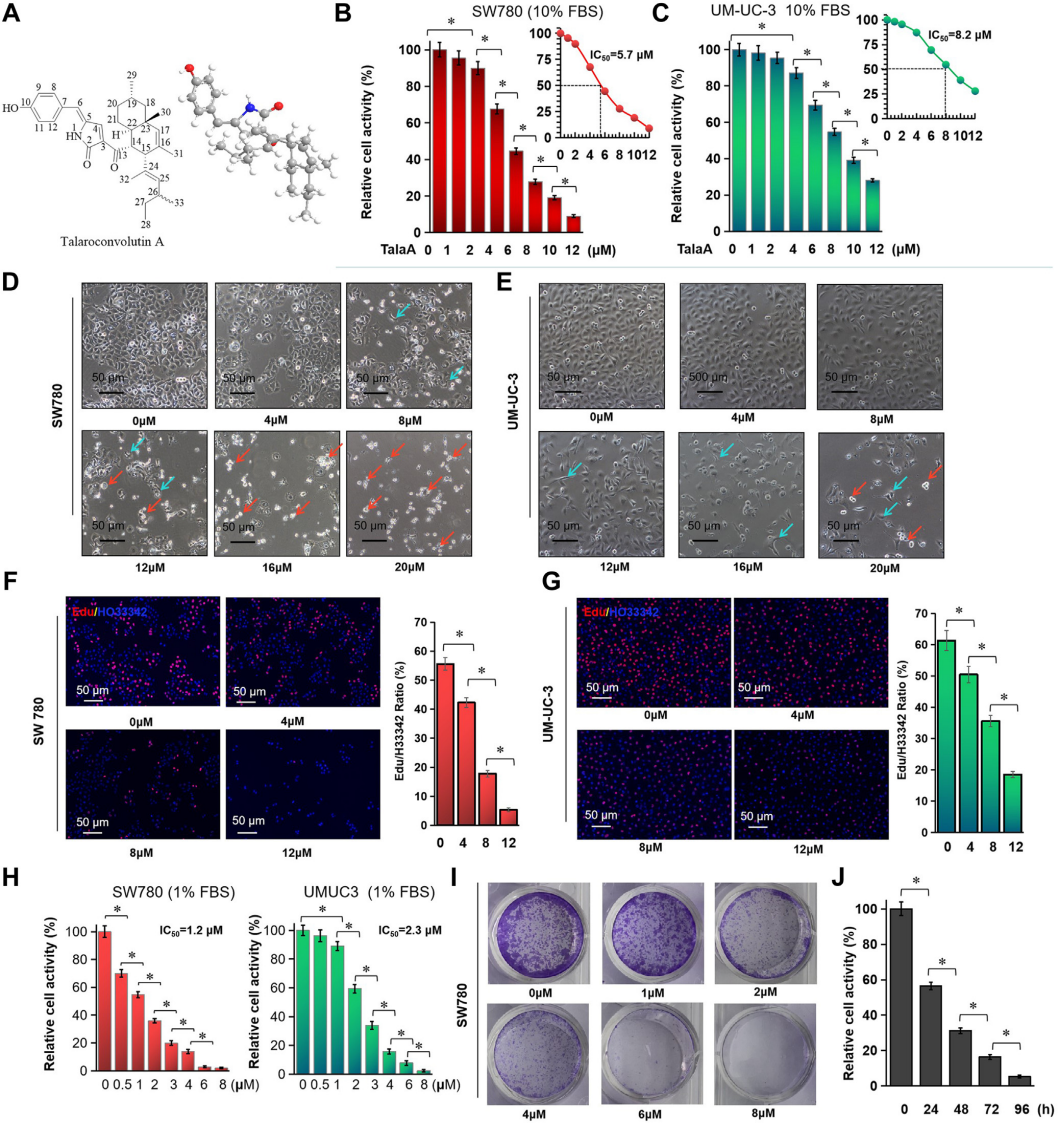
**TalaA inhibited bladder cancer cells growth dose- and time-dependently *in vitro*.***A*, the chemical formula of TalaA. *B* and *C*, the bladder cancer cells were treated by 0 to 12 μM TalaA and the activity was examined with CCK8 kit. In cell culture media containing 10% FBS, the IC_50_ of TalaA for SW780 and UM-UC-3 is 5.7 and 8.2 μM, respectively. The difference significance between two groups was calculated by one-way ANOVA followed by LSD post hoc analysis, ∗*p* < 0.05. *D* and *E*, bladder cancer SW780 and UM-UC3 cells were co-incubated with 0 to 20 μM TalaA for 24 h. The cells’ morphological alteration was recorded by microscope. *Blue arrows* represented the cells became narrow and long; *red arrows* represented cells lose their extensibility and tend to detach from the plate. *F* and *G*, cells were treated by 0 to 12 μM TalaA, and EdU kit was employed to detect DNA synthesis speed. *Red spots* represented EdU-positive stained cells, and *blue spots* represented nuclei. The difference significance between two groups was calculated by one-way ANOVA followed by LSD post hoc analysis, ∗*p* < 0.05. *H*, the bladder cancer cells were treated by 0 to 8 μM TalaA and the activity was determined by CCK8 kit. In cell culture media containing 1% FBS, the IC_50_ of TalaA for SW780 is 1.2 μM and IC_50_ of TalaA for UM-UC-3 is 2.3 μM. The difference significance between two groups was calculated by one-way ANOVA followed by LSD post hoc analysis, ∗*p* < 0.05. *I*, bladder cancer SW780 cells were co-incubated with 0 ∼ 8.0 μM TalaA for 10 days, and the cells was stained by crystal violet. *J*, SW780 cells were treated by 5.0 μM TalaA for 24, 48, 72, and 96 h. The cell activities were examined by CCK8. ∗*p* < 0.05. EdU, 5-Ethynyl-2′-deoxyuridine; FBS, fetal bovine serum; TalaA, talaroconvolutin-A.

The chemical structure of TalaA is shown in Figure 1*A*. As shown in Figure 1, *B* and *C*, TalaA inhibited the proliferation of SW780 and UM-UC-3 cells with IC_50_ values of 5.7 and 8.2 μM, respectively (experiments performed in DMEM containing 10% FBS). Morphological changes in bladder cancer cells caused by TalaA are shown in Figure 1, *D* and *E*. TalaA dose dependently decreased cell density and led to morphological alterations in SW780 and UM-UC-3 cells. SW780 was more vulnerable than UM-UC3 to the effects of TalaA; 8 μM TalaA caused SW780 cells to become narrow and long (blue arrow); 12 μM TalaA led SW780 cells to lose their extensibility and detach from the plate (red arrow). The ratio of EdU/H33342 can reflect the DNA replication rate. We used EdU to determine the inhibitory effect of TalaA on DNA replication. As shown in Figure 1, *F* and *G*, as the concentration of TalaA increased, the EdU-positive ratio decreased rapidly, indicating that TalaA slowed DNA replication in bladder cancer cells. Moreover, the inhibitory effect of TalaA on bladder cancer was examined at low serum concentrations. SW780 and UM-UC-3 cells were more sensitive to TalaA in a 1% FBS medium than in a 10% FBS medium (Fig. 1*H*). In the presence of low basal levels of serum (1% FBS), the IC_50_ values of SW780 and UM-UC-3 were 1.2 and 2.3 μM, respectively.

### TalaA Inhibited Bladder Cancer Cell Growth *in Vitro* in a Time-Dependent Manner

Colony formation experiments can detect the inhibition of long-term drug incubation on the proliferation of cancer cells. The colony formation assay showed that TalaA inhibited the clonogenic ability of SW780 cells (Fig. 1*I*). Notably, 6.0 μM TalaA almost completely inhibited colony formation (Fig. 1*I*), whereas this concentration of TalaA inhibited cell proliferation by less than 40% (Fig. 1*B*). This was because, in the colony formation experiment, the incubation time of TalaA was 10 days, whereas, in the cell proliferation experiment, the TalaA treatment time was 24 h. This result indicates that the TalaA treatment time is related to its cancer inhibitory effect. As shown in Figure 1*J*, as the TalaA incubation time increased, the residual viability of bladder cancer cells decreased, indicating that TalaA suppressed bladder cancer growth in a time-dependent manner.

### TalaA Suppressed Bladder Tumor Growth *in Vivo* in a Xenografted Mouse Model

**Fig. 2:**
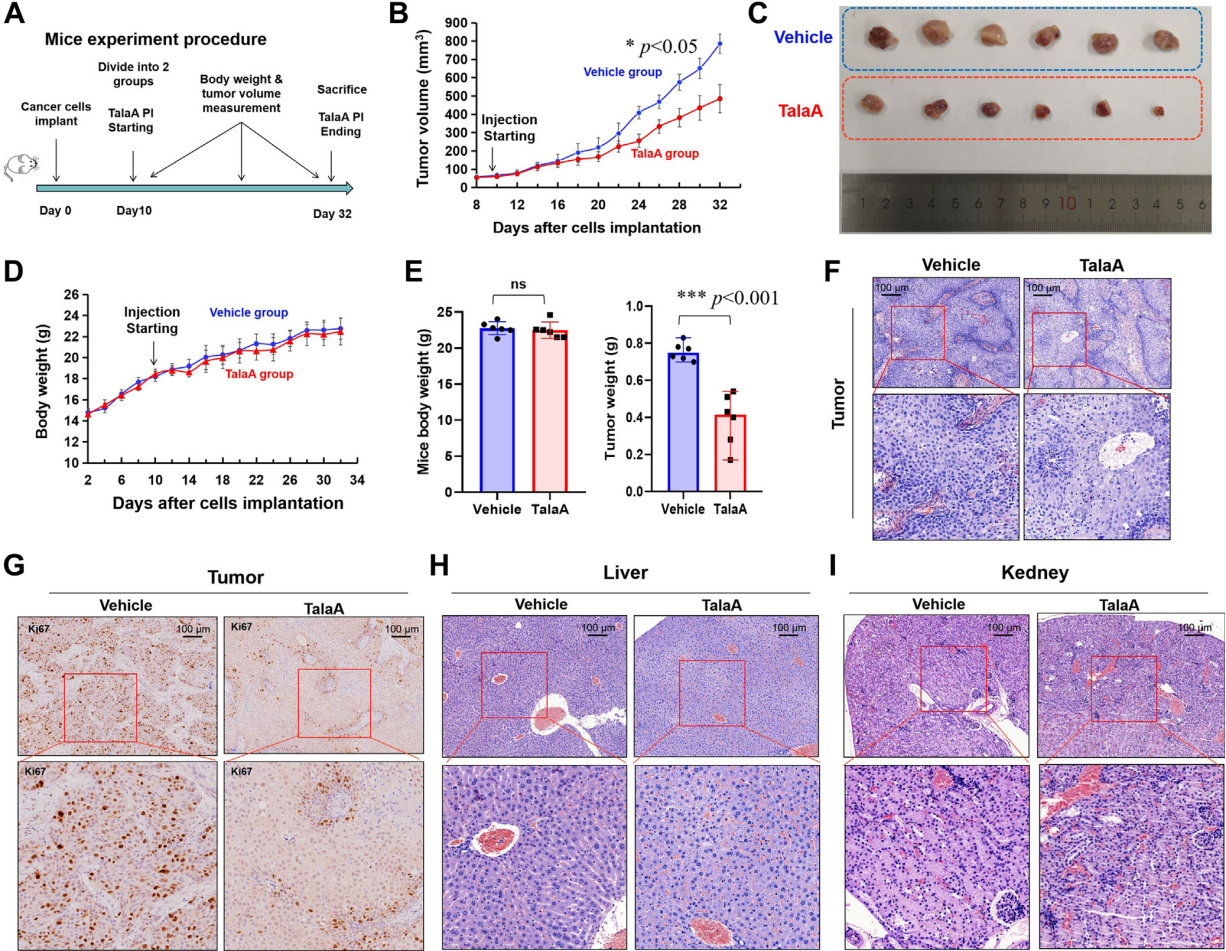
**TalaA inhibited bladder tumor growth *in vivo* with low toxicity.***A*, the time schedule diagram of xenografted tumor and TalaA injection in mice model. *B*, the tumor sizes of TalaA-treated group and vehicle control group were calculated and recorded. The difference significance between two groups was calculated by Student's *t* test, ∗*p* < 0.05. *C*, photos of xenografted tumor peeled from subcutaneous tissue of mice. The *upper row* represents the vehicle control group, and the *lower row* represents the TalaA-treated group. *D*, the mice body weight of TalaA-treated group and vehicle control group were calculated and recorded. *E*, after mice were sacrificed, the mice body weight and tumor weight was compared between control group and TalaA treatment group. The difference significance between two groups was calculated by Student's *t* test, ∗∗∗*p* < 0.001; ns: no significant difference. *F*, H&E-stained transplanted tumor. *Left*, vehicle control mice; *right*, TalaA-treated mice. *G*, IHC staining of proliferation marker ki67 for transplanted tumor. *H*, H&E-stained mice liver. *Lower* pictures are from *upper* ones. *I*, H&E-stained mice kidney. *Lower* pictures are from *upper* ones. IHC, immunohistochemistry; TalaA, talaroconvolutin-A.

Modeling of the administration procedure of subcutaneous tumors in mice was shown in Figure 2*A*. The growth rate of transplanted tumors in the TalaA treatment group was significantly lower than that in the control group (Fig. 2*B*). Figure 2*C* showed that the tumor volume of the TalaA treatment group was significantly smaller than that of the control group. Figure 2*D* shows the changes in the body weight of mice in the TalaA treatment and control groups. TalaA did not significantly affect the body weight of mice. We next quantitatively compared the body and tumor weights of mice in the two groups. As shown in Figure 2*E*, TalaA significantly reduced the subcutaneous tumor weight but did not significantly affect the body weight of mice. H&E and IHC staining (Fig. 2, *F* and *G*) showed that TalaA significantly decreased the expression of the marker protein Ki67, indicating that TalaA can repress cell proliferation in transplanted tumors. H&E staining did not reveal obvious abnormalities in the liver and kidney, suggesting that TalaA has no serious toxicity in the liver and kidney (Fig. 2, *H* and *I*).

### TMT-Labeled Quantitative Proteomics Results Indicated that TalaA Mainly Targeted the Cell Cycle Pathway in Bladder Cancer Cells

**Fig. 3:**
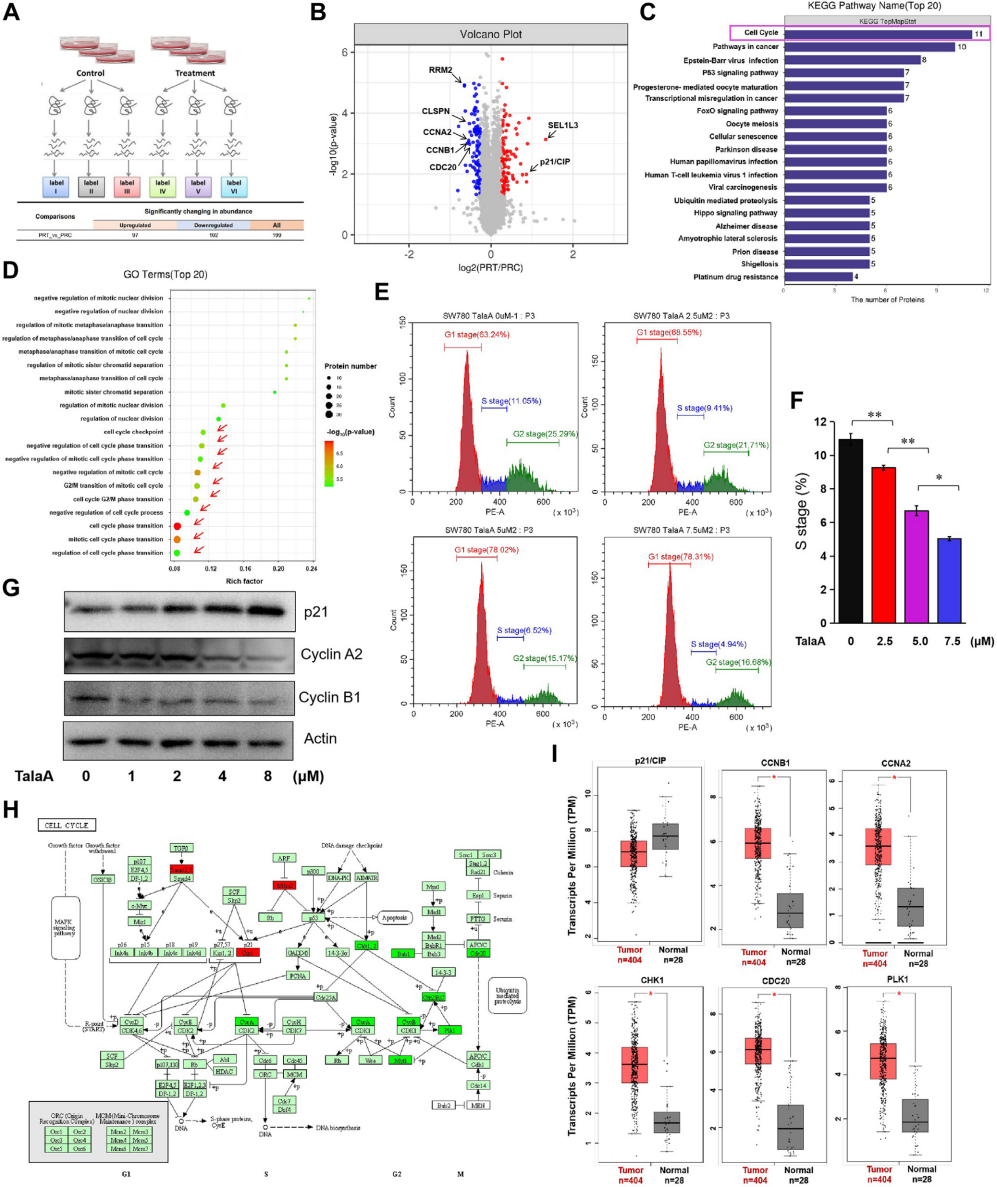
**Proteomic results indicated TalaA-repressed cell cycle of bladder cancer cells.***A*, schematic diagram of proteomics experiment process. The concentration of TalaA for this experiment is 8.0 μM. *B*, volcano plot of differential proteins between TalaA treatment group and control group. *C*, KEGG enrichment analysis was performed for differential proteins, and the top 20 pathways ranked according to significance were listed in the figure. *D*, GO enrichment analysis was performed for differential proteins, and the top 20 pathways were listed in the figure. *E*, the cell cycle was regulated by TalaA in SW780 bladder cancer cells. *F*, TalaA reduced S phase of cell cycle in bladder cancer SW780 cells. The difference significance between two groups was calculated by one-way ANOVA followed by LSD post hoc analysis, ∗*p* < 0.05, ∗∗*p* < 0.01. *G*, Western blotting data showed that the TalaA elevated cell cycle check proteins p21/CIP and decreased cell cycle promoting proteins CCNB1 and CCKA2 in bladder cancer SW780 cells. *H*, the TalaA-caused changes of cell cycle-related proteins were shown in the KEGG map. The *red boxes* represented upregulated proteins and the *green boxes* represented downregulated proteins. *I*, the TalaA-affected P21/CIP, CCNB1, CCNA2, CHK1, CDC20, and PLK1 were significantly changed in clinical bladder cancer patients (data were from GEPIA http://gepia.cancer-pku.cn/). GO, gene ontology; KEGG, Kyoto Encyclopedia of Genes and Genomes; TalaA, talaroconvolutin-A.

To reveal the molecular mechanism by which TalaA represses bladder cancer, we analyzed the protein alterations caused by TalaA in TMT-labeled quantitative high-throughput proteomics. A total of 49,639 peptides were identified, among which 6716 proteins were identified. Figure 3*A* shows an overview of the proteomic workflow. As shown at the bottom of Figure 3*A*, TalaA significantly elevated 97 proteins and repressed 102 proteins in bladder cancer cells. Proteomic changes induced by TalaA are shown in the volcano diagram in Figure 3*B*, indicating that TalaA downregulated proteins promoting the cell cycle (such as cyclin A2 (CCNA2), cyclinB2 (CCNB1), cell division cycle 20 (CDC20), and ribonucleotide reductase subunit M2 (RRM2)) and upregulated those suppressing the cell cycle (such as p21/CIP). To identify the primary pathways affected by TalaA, KEGG and GO enrichment analyses were performed. Figure 3*C* shows the top 20 pathways according to significance, among which the top-ranked pathway was “cell cycle”. Moreover, GO enrichment analysis showed that the top 20 “biological process” pathways were comprehensively ranked in the GO analysis results and belonged to cell cycle–related pathways, including the cell cycle checkpoint, nucleus division, and mitosis transition pathways (Fig. 3*D*). The flow cytometry results (Fig. 3, *E* and *F*) showed that TalaA suppressed the cell cycle of bladder cancer cells, which is consistent with the results of the enrichment assay using the proteomics data. To verify the proteomics results, we performed Western blotting to evaluate the expression levels of cell cycle–related proteins. As shown in Figure 3*G*, TalaA increased p21/CIP but reduced CCNA2 and CCNB1 at the protein level, which agrees with the proteomics data. These results indicate that TalaA alters cell cycle–related molecules at the protein level, thereby suppressing bladder cancer cell proliferation. To graphically evaluate the effects of TalaA on the cell cycle, we constructed the KEGG cell cycle pathway (Fig. 3*H*). The green boxes in the figure represent decreased molecules, and the red box represents increased molecules. The results showed that TalaA inhibited cell cycle by increasing cycle check point protein p21/CIP, while reducing cyclin A (CCNA), cyclin B (CCNB), cell division cycle 20 (CDC20), myelin transcription factor 1 (MYT1), checkpoint kinase 1 (CHK1), polo-like kinase 1 (PLK1), and other cell cycle–related proteins. Next, we compared the expression of these genes in clinical patients using an online database (http://gepia.cancer-pku.cn/). As shown in Figure 3*I*, these genes were abnormally expressed in bladder cancer patients. Notably, this abnormal expression was salvaged by TalaA, suggesting that TalaA inhibits the proliferation of bladder tumor cells by regulating abnormal cell cycle–related proteins.

### TalaA-Triggered Bladder Cancer Cell Death Through ROS-Involved Ferroptosis

**Fig. 4:**
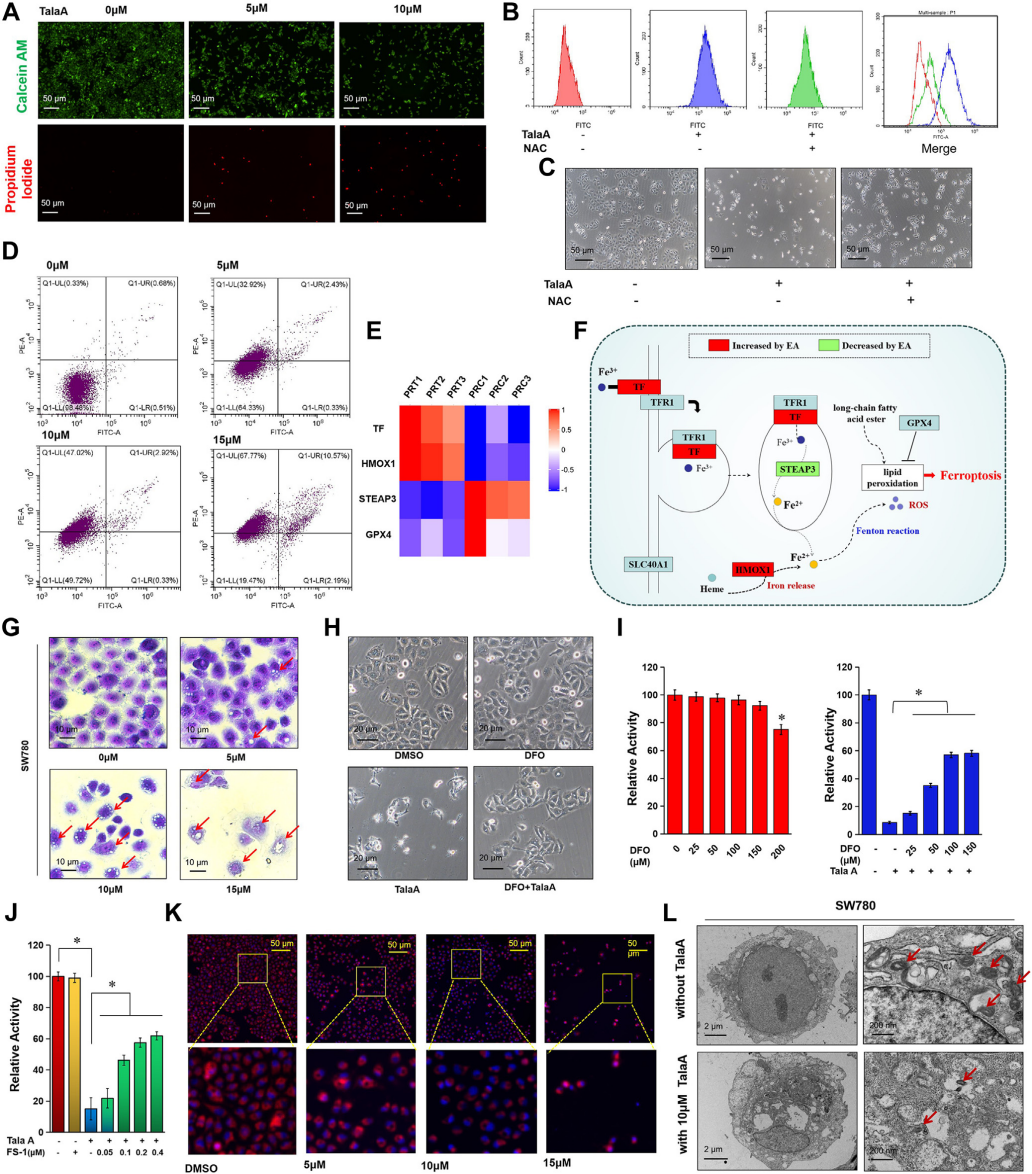
**TalaA induced bladder cancer cell death through not apoptosis but ferroptosis.***A*, “living/dead cell” staining for TalaA-treated SW780 cells. The calcein-AM–stained cells in *green* represented living cells, and PI stained in *red* represented dead cells. *B*, the intracellular ROS was detected by H2DCFDA. TalaA induced ROS in SW780 cells, and the ROS could be neutralized by NAC. The TalaA concentration was 10 μM, and NAC concentration was 1 mM in the experiment. *C*, the cell morphological evidence of NAC rescuing TalaA-induced cell death in SW780 cells. *D*, cell apoptosis was detected by flow cytometry. Bladder cancer SW780 cells were co-incubated with 0 to 12 μM TalaA for 48 h. Annexin V-FITC/PI was used to test the apoptosis. *E*, the effect of TalaA on HMOX1, TF, and STEAP3 protein levels was shown in Heatmap. PRT represented TalaA treatment group; PRC represented DMSO control group. *Red* represented upregulation, and *blue* represented downregulation. *F*, the TalaA-caused changes of ferroptosis-related proteins were shown in the schematic diagram. The *red boxes* represented upregulated proteins and the *green boxes* represented downregulated proteins. *G*, 0 to 12 μM TalaA-treated SW780 cells were stained by crystal violet. TalaA induced vacuolation of SW780 cells in a concentration-dependent manner. *H*, morphological evidence of deferiprone (DFO) rescuing TalaA-induced cells death. The SW780 cells pretreated with 100 μM DFO for 1 h were co-incubated with 12 μM TalaA for 24 h. *I*, *left*, cell activity of SW780 cells incubated with DFO for 24 h, ∗*p* < 0.05. *Right*, cell activity of SW780 cells incubated with DFO and TalaA for 24 h, ∗*p* < 0.05. DFO rescued TalaA-suppressed SW780 cell activity, ∗*p* < 0.05. *J*, ferroptosis inhibitor ferrostatin-1 (FS-1) rescued TalaA-repressed bladder cancer SW780 cell activity, ∗*p* < 0.05. *K*, the mitochondrial membrane potential detection. *Red* represented Mito-tracker Red CMXRos-stained mitochondria and blue represented Hoechst 33342-stained nuclei. *L*, transmission electron micrograph of SW780 cells treated with TalaA. The *red arrows* represented the mitochondria. DMSO, dimethyl sulfoxide; HMOX1, heme oxygenase 1; NAC, N-Acetyl-L-cysteine; ROS, reactive oxygen species; TalaA, talaroconvolutin-A; TF, transferrin; .

TalaA treatment decreased the proportion of living cells and increased that of dead cells, indicating that TalaA causes cell death (Fig. 4*A*). Using the ROS probe H_2_DCFDA, we found that TalaA increased intracellular ROS levels (Fig. 4*B*). Morphological experiments showed that the ROS scavenger N-Acetyl-L-cysteine rescued TalaA-induced cell death, suggesting that TalaA induces cell death through ROS (Fig. 4*C*). Flow cytometry was performed to determine whether TalaA induces apoptosis. As shown in Figure 4*D*, TalaA did not cause bladder cancer cells to move from quadrant LL to LR but moved from LL to UL, indicating that TalaA did not induce obvious apoptosis but induced cell death in a nonapoptotic manner. We previously showed that TalaA induces ferroptosis in colorectal cancer cells. Our current proteomic data shows that TalaA caused significant changes in ferroptosis pathway–related proteins (Fig. 4*E*). The roles of these key proteins are shown in a KEGG signaling pathway map (Fig. 4*F*), and the results suggest that TalaA induced cell death *via* ferroptosis. The morphological features of ferroptosis include cell surface perforation, decreased mitochondrial membrane potential, mitochondrial shrinkage, and ridge disappearance, which were examined in bladder cancer cells. Crystal violet staining revealed TalaA-induced perforation on the surface of bladder cancer cells (Fig. 4*G*). As shown in Figure 4*H*, chelator DFO significantly reduced TalaA-induced morphological abnormalities in SW780 cells. Furthermore, DFO rescued TalaA-induced cell inactivity in a concentration-dependent manner (Fig. 4*I*). Additionally, another ferroptosis inhibitor ferrostatin-1 dose-dependently rescued TalaA-caused cell inactivity (Fig. 4*J*), indicating TalaA was able to induce ferroptosis. The mitochondrial membrane potential decreased gradually with increasing TalaA concentrations (Fig. 4*K*). Transmission electron microscopy images showed that TalaA led to mitochondrial shrinkage, and the ridges in the mitochondria decreased or disappeared significantly (Fig. 4*L*), which is a typical morphological characteristic of ferroptosis. These results suggest that TalaA induces bladder cancer cell death through ferroptosis rather than through apoptosis.

### Transcriptional Changes of Bladder Cancer Cells Induced by TalaA

**Fig. 5:**
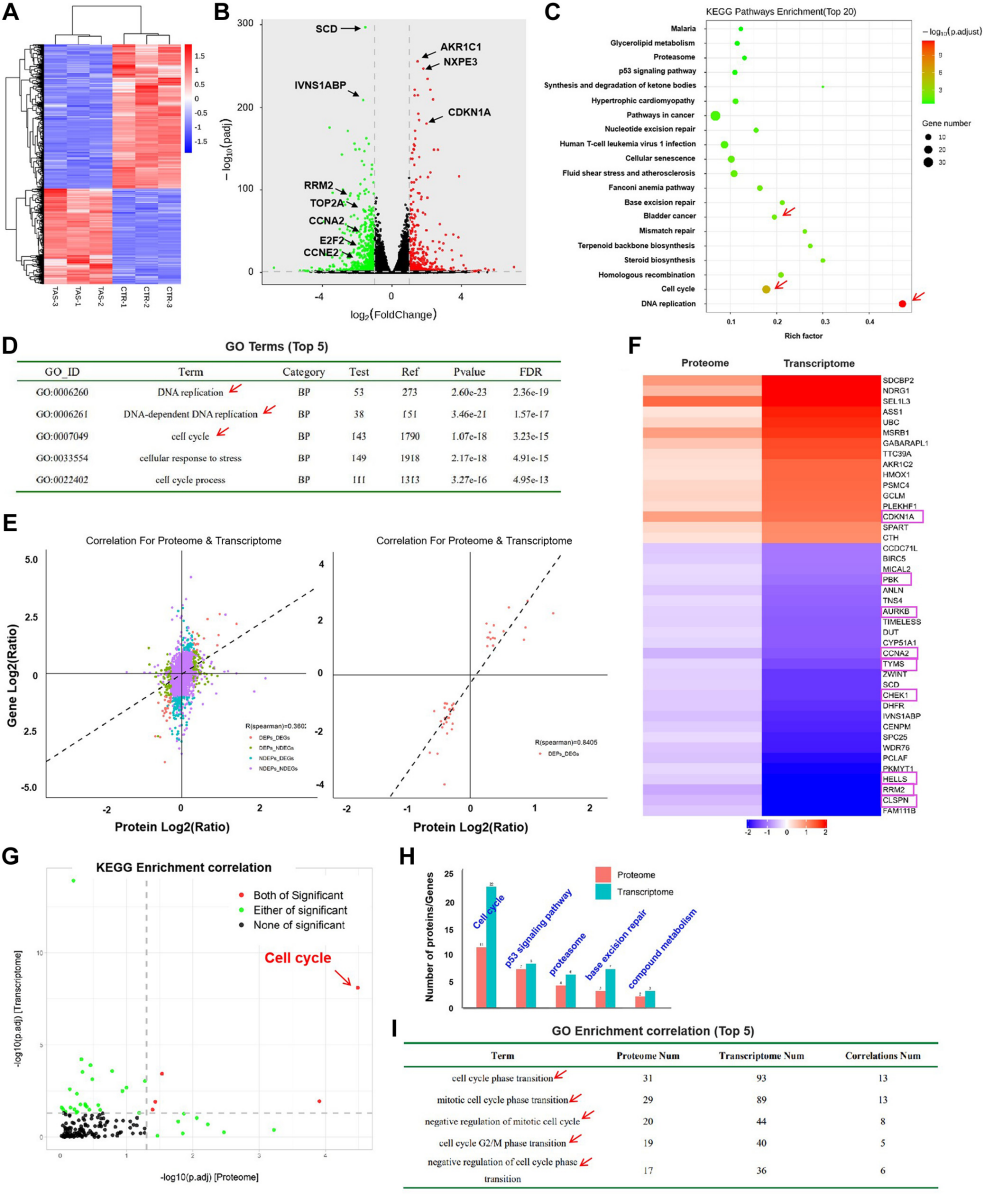
**Transcriptomics and proteomics analysis verified that TalaA mediated the transcription of cell cycle–related molecules.***A*, heatmap results for TalaA on the transcriptome of bladder cancer cells. CTR represented the control group and TAS represented the TalaA treatment group. *B*, volcano plot of differential genes between TalaA treatment group and control group. *Green spots* represented downregulated genes, and *red spots* represented upregulated genes. *C*, KEGG enrichment analysis was performed for differential transcripts, and the top 20 pathways ranked according to significance were listed in the figure. *D*, the first five pathways of biological process GO enrichment analysis results. *E*, the correlation plot between TalaA-induced differential proteins and differential mRNAs. DEPS represented differential expressed proteins and NDEPS represented nondifferential proteins; DEGS represented differential expressed genes and NDEGS represented nondifferential genes. *F*, this map listed the genes that are consistent with the transcriptional level and protein level in bladder cancer cells after TalaA treatment. Cell cycle–related genes were labeled by *rose-red boxes*. *G*, after KEGG enrichment analysis, the differential molecules in both proteome and transcriptome were taken the intersection. There were five pathways with significant changes in the intersection (*red dots*), which cell cycle was the most significant, and the fold change was the largest. *H*, combining proteome and transcriptome, the top five pathways with the largest number of differential molecules were as follows: cell cycle, p53 signaling, proteasome, base excision repair, and compound metabolism pathways. *I*, through GO enrichment analysis of differential molecules between proteome and transcriptome, the top five in the number of differential molecules and correlation differential molecules was summarized. Note that all of them are cell cycle–related pathways. GO, gene ontology; KEGG, Kyoto Encyclopedia of Genes and Genomes; TalaA, talaroconvolutin-A.

We performed transcriptomic analysis to further study this mechanism. Figure 5*A* shows a heatmap of the transcriptome differential molecules between the TalaA treatment and control groups. As shown in the volcano map, TalaA significantly decreased the expression of cell cycle–promoting genes (TOP2A, RRM2, CCNB1, and CCNA2) and increased the expression of cell cycle–suppressing genes (such as CDKN1A) (Fig. 5*B*). Next, KEGG enrichment analysis was performed, and the top 20 pathways were listed. As shown in Figure 5*C*, the top two KEGG enrichment analysis results were “cell cycle” and “DNA replication.” Consistently, the top three GO enrichment analysis results were the “DNA replication” and “cell cycle” pathways (Fig. 5*D*). These results suggest that TalaA regulates cell cycle–related molecules at the transcriptional level.

### Combined Analysis of Transcriptome and Proteome Indicated TalaA Mainly Targeted Cell Cycle–Related Molecules

We performed a combined analysis of the transcriptome and proteome and identified a group of genes showing consistent alterations at both the transcriptional and protein levels (Fig. 5*E*). Cell cycle–related genes (*CDKN1A*, *PBK*, *AURKB*, *CCNA2*, *TYMS*, *CHEK1*, *HELLS*, *RRM2*, and *CLSPN*) showed the same trend alterations at both the transcription and protein levels (Fig. 5*F*). We drew a four-quadrant diagram of the correlated genes, which showed that the cell cycle pathway was the most significant in the correlated KEGG pathways (Fig. 5*G*). Furthermore, we counted the number of differentially expressed genes in both the transcriptome and proteome. Figure 5*H* shows that the cell cycle pathway ranked highest among the top five KEGG pathways. Moreover, in the correlation assessment of the GO enrichment results, the top five pathways were all cell cycle–related pathways (Fig. 5*I*). These results indicate that TalaA mainly targets DNA replication– and cell division–related genes to inhibit bladder cancer.

### TalaA Induced Phosphoproteomics Alteration by Affecting Kinase Activity

**Fig. 6:**
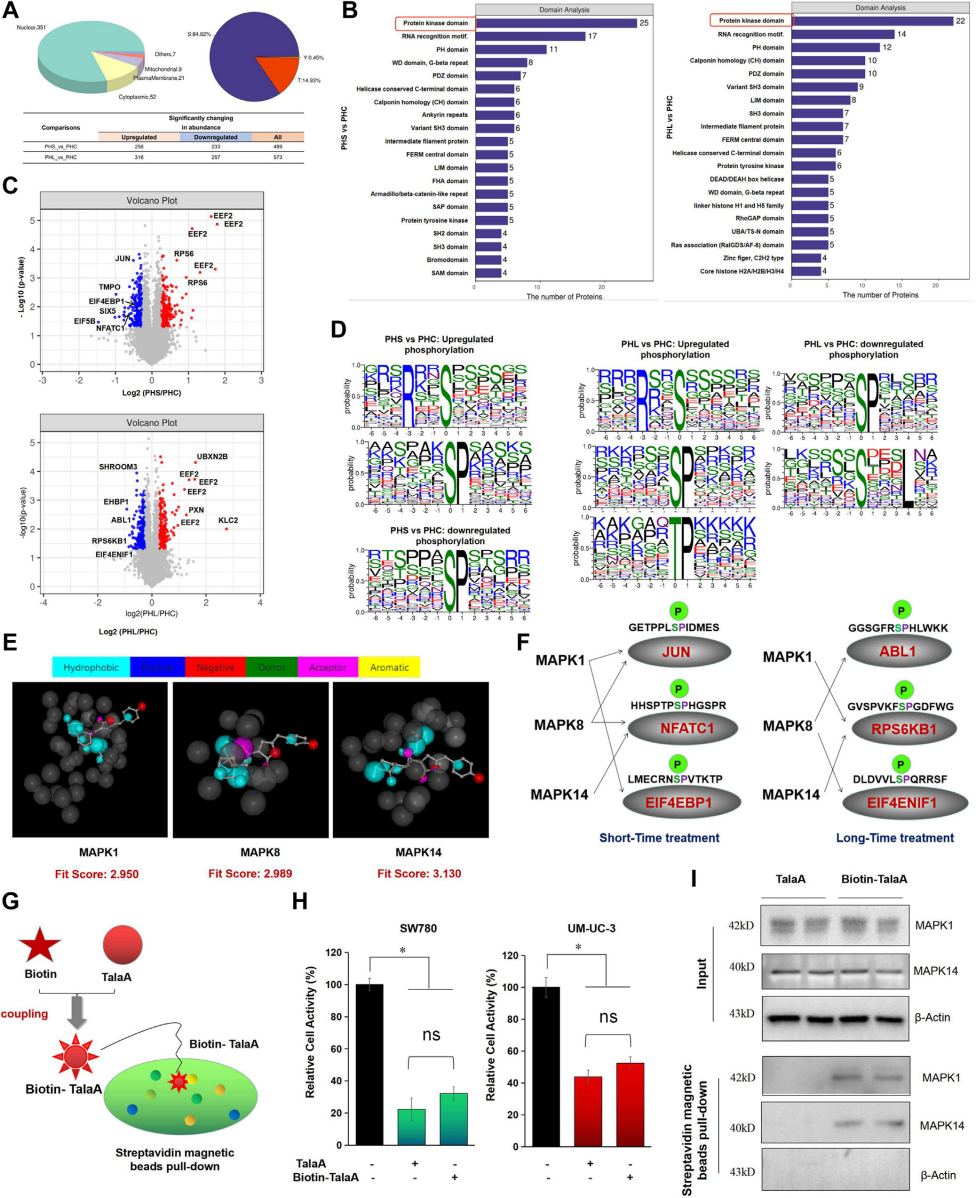
**TalaA induced the phosphoproteomics alteration.***A*, subcellular localization (*left*) and phosphorylation site (*right*) of phosphomics alteration induced by TalaA. In the inserted table, PHS represented shorter-time treatment (10 min), PHL represented longer-time treatment (30 min), and PHC represent without TalaA treatment. *B*, enrichment analysis of protein domains with significant phosphorylation changes. The *red box* indicated the enrichment results with the highest ranking. *C*, volcano plot of differential genes between TalaA treatment group and control group. *Blue spots* represented downregulated phosphorylation site, and *red spots* represented upregulated phosphorylation site. *D*, motif of phosphorylation sites affected by TalaA. In the X-axis, the zero point is the phosphorylated amino acid, and its upstream and downstream amino acids are the neighbor amino acid. And the Y-axis indicates probability. The *larger letter* represents the higher probability. *E*, three dimensional diagram of interaction structure between TalaA and MAPKs. The *colored atoms* in the diagram represented TalaA, and the interaction atoms of MAPKs were displayed by *dark gray*. *F*, the diagram of MAPKs on the substrate with “∗SP∗” motif site. The *green* S represented the phosphorylation site, and P represented the phosphate group. *G*, the diagram of biotin-coupling TalaA and the pulldown experiment using Biotin-TalaA. *H*, inhibitory effects of original TalaA and Biotin-TalaA on bladder cancer cells SW780 and UM-UC-3, ∗*p* < 0.05. *I*, Western blotting data for Biotin-TalaA-pulldown components (*lower* figures) and the input (*upper* figures). MAPK, mitogen-activated protein kinase; TalaA, talaroconvolutin-A.

To further examine the pharmacological mechanism of TalaA, phosphoproteomics was performed. A total of 4122 phosphorylated proteins were identified, among which 4122 modified proteins had 15,425 quantitative phosphorylated peptides and 21,622 quantitative phosphorylated sites. As shown in Figure 6*A*, shorter-term treatment caused significant changes in 489 phosphorylated proteins, while longer-term treatment led to significant alteration in 573 phosphorylated proteins. TalaA affected the phosphorylation, including hundreds of nuclear proteins’ phosphorylation. Among them, serine phosphorylation accounted for the largest percentage (84.62%), whereas tyrosine phosphorylation accounted for only 0.45% of TalaA-induced phosphorylation alterations (shown in Fig. 6*A*). Analysis of the protein domains of all proteins showing significant phosphorylation changes showed that the protein kinase domain was ranked first (Fig. 6*B*). Volcanic maps were used to visualize the phosphorylation changes caused by TalaA. As shown in Figure 6*C*, most phosphorylated proteins downregulated by TalaA are important transcription factors in cancer cells (*e.g.*, JUN, NFATC1), translation regulatory elements (eukaryotic translation initiation factor 4E binding protein 1 (EIF4EBP1) and EIF4ENIF1), or important signal transduction molecules of cancer cells (*e.g.*, ABL1, ribosomal protein S6 kinase B1 (RPS6KB1)); the phosphorylated protein exhibiting the most significant upregulation was EEF2 at Thr57 and Thr59. To explore the features of TalaA-induced phosphorylation changes, we statistically summarized all altered phosphorylation sites and drew the motif (Fig. 6*D*). As shown in Figure 6*D*, alterations in TalaA-induced phosphorylation mainly occurred at the serine site in “∗SP∗” sequences. Next, we screened the potential proteins that interact with TalaA using the molecular interaction prediction website PharmMapper (http://lilab-ecust.cn/pharmmapper/) and found that TalaA tended to interact with kinases, the most important of which were mitogen-activated protein kinases (MAPKs). Table 1 shows the predictions of the interaction parameters between TalaA and MAPKs. Figure 6*E* shows the visualized molecular models of TalaA interactions with MAPK1, MAPK8, and MAPK14. Based on our phosphoproteomic analysis results and the reported relationship between MAPKs and their substrates (*e.g.*, JUN and ABL1), we drew a relationship diagram of MAPKs affecting protein phosphorylation (Fig. 6*F*). These results indicate that TalaA inactivates bladder cancer–related transcription factors (*e.g.*, JUN, EIF4EBP1, and RPS6KB1) by inhibiting the phosphorylation of multiple MAPKs. To verify the interaction between TalaA and MAPKs, the streptavidin magnetic bead pulldown experiment was performed (Fig. 6*G*). The coupled Biotin-TalaA was verified and shown in Supplementary Materials. The antitumor activity of Biotin-TalaA was verified by CCK-8 assay. The results showed that TalaA still had anticancer activity after coupling with Biotin, indicating that Biotin did not destroy the anticancer active sites of TalaA (shown in Fig. 6*H*). After streptavidin magnetic bead-pulldown experiment, Western Blotting was performed to check the TalaA-binding molecules. As shown in Figure 6*I*, The Biotin-TalaA group can pulldown MAPK1 and MAPK14, which indicates that TalaA interacts with the above MAPKs.

**Table 1:** Prediction of the interaction between TalaA and MAPKs

Pharma model	Num feature	Fit	Norm Fit	Name
1pme_v	3	2.95	0.9833	Mitogen-activated protein kinase 1, MAPK1 (ERK2)
1uki_v	3	2.989	0.9963	Mitogen-activated protein kinase 8, MAPK8 (JNK1)
1bl6_v	4	3.13	0.7826	Mitogen-activated protein kinase 14, MAPK14

## Discussion

TalaA is a secondary metabolite in the endophytic fungus *T. purpurogenius*; its biological activity, particularly its antitumor effects, is not well-understood. Only one article was published in 2021, which reported that TalaA kills colorectal cancer cells mainly by inducing ferroptosis. We recently demonstrated that TalaA exerts anticancer functions in bladder cancer. Interestingly, the pharmacological mechanism of TalaA in bladder cancer differs from that in colorectal cancer. In bladder cancer, TalaA mainly inhibits cell proliferation by blocking DNA replication and inhibiting the cell cycle. TalaA causes bladder cancer cell death through ferroptosis rather than through apoptosis. TalaA elevates intracellular ROS and upregulates ferroptosis enhancers in bladder cancer cells. In contrast to our previous findings, TalaA-triggered ferroptosis of bladder cancer cells does not occur through the inhibition of SLC7A11 expression but rather through the upregulation of transferrin and heme oxygenase 1 in bladder cancer cells.

The molecular mechanism of bladder cancer inhibition by TalaA was analyzed using multi-omics approaches, including proteomics, transcriptomics, and phosphoproteomics, and verified experimentally. TalaA mainly targeted the cell cycle of bladder cancer cells, particularly in the S stage of the cell cycle. Cyclins, cyclin-dependent kinases (CDKs), and CDK inhibitors are key molecules regulating the cell cycle. Periodic accumulation and decomposition of cyclins play important roles in the cell cycle, whereas CDK inhibitors negatively regulate the cell cycle by inhibiting CDK. We found that TalaA blocked the transition from the G1 to S phase of bladder cancer cells and downregulated CCNA2 and CCNB1, which are well-known therapeutic targets for various cancers (11, 12, 13). Additionally, TalaA upregulated p21 at both the transcriptional and protein levels and thereby inhibited DNA replication in cancer cells. P21/CIP is a cyclin-dependent kinase inhibitor whose NH_2_ terminus can recognize the cyclin–CDK complex, preventing phosphorylation of the threonine site of CDK and inhibiting its kinase activity, thus blocking the progression of the G1 to S phase of the cell cycle (14).

Abnormal protein phosphorylation is a common feature of malignant tumor cells (15). Many targeted drugs for tumor therapy (*e.g.*, tepotinib and crizotinib) that target the phosphorylation of key proteins in tumor cells have been developed (16, 17). To understand the molecular pharmacological mechanisms of TalaA against bladder cancer, we analyzed changes in TalaA-induced phosphorylation. TalaA downregulated the phosphorylation of a series of transcription factors, including JUN, NFATC1, EIF4EBP1, ABL, RPS6KB1, and EIF4ENIF1, most of which play pivotal roles in cell proliferation and tumor progression (18, 19, 20). We also evaluated the relationship between the antitumor mechanism and inhibition of phosphorylation of JUN, EIF4EBP1, and RPS6KB1.

C-Jun (a translation product of *JUN*) is an oncogenic transcription factor that interacts with c-Fos to form the AP-1 early reaction transcription factor (21). C-Jun exerts numerous important functions, including proliferation, apoptosis, survival, and morphogenesis (22). Classical studies showed that c-Jun is closely related to cell proliferation. Phosphorylation of c-Jun establishes a molecular link between growth factor signaling and cell cycle regulators, which can regulate cell cycle–related proteins (such as CCNA2 and CCND1) through transcriptional regulation to promote the cell cycle, particularly the transition from G1 to S phase (23, 24). Downregulation of c-Jun phosphorylation by TalaA may be targeted for developing anticancer drugs.

EIF4EBP1 is a member of the protein translation inhibitor family. This protein directly interacts with eukaryotic translation initiation factor 4E (eIF4E) to control translation (25). MAPK phosphorylates EIF4EBP1, which binds to the 5′-cap of eIF4E mRNA and inhibits cap-dependent translation (26). EIF4EBP1 is phosphorylated in response to various signals, causing it to dissociate from eIF4E and activate mRNA translation (25). High expression of EIF4EBP1 is associated with poorer survival and worse disease progression in patients with hepatocellular carcinoma (27). We found that phosphorylation of EIF4EBP1 was significantly reduced in the TalaA-treated group, indicating that TalaA inhibits translation in bladder cancer cells by downregulating EIF4EBP1 phosphorylation.

RPS6KB1 regulates diverse cellular processes, including cell growth, motility, survival, and proliferation. RPS6KB1 phosphorylation is related to worse prognosis in patients with nonsmall cell lung cancer (28). Wang-Bishop *et al.* (29) reported that inhibition of phosphorylated RPS6KB1 at T389 in KRAS mutant gastrointestinal cancer cells effectively inhibited cell proliferation and suppressed the growth of xenografted tumors in a mouse model. Our findings suggest that TalaA-attenuated RPS6KB1 phosphorylation would contribute to inhibit bladder cancer.

Enrichment analysis of the phosphoproteomic results showed that protein kinase ranks first among all molecules with significant phosphorylation changes caused by TalaA treatment. TalaA tended to combine with various MAPKs (*e.g.*, MAPK1, MAPK8, and MAPK14). The motifs of TalaA-affected phosphorylation sequences matched the substrate sequence motifs of MAPKs. Moreover, TalaA affected the phosphorylation motif of important transcription factors (*e.g.*, c-Jun) or transcription regulators (*e.g.*, EIF4EBP1), playing a tumor-promoting role *via* phosphorylation. These findings suggest that TalaA interacts with MAPKs to regulate the phosphorylation of various transcription factors, translation regulatory elements, and signal transduction molecules, thus playing an antitumor role.

In summary, TalaA inhibited bladder cancer mainly by arresting the cell cycle to suppress proliferation and triggering ferroptosis to cause cell death. TalaA primarily targets cell cycle–related pathways (particularly DNA replication). TalaA inhibits protein kinase activity by interacting with MAPKs, resulting in decreased phosphorylation of downstream substrates, including important transcription factors and translation regulatory elements, thereby suppressing bladder cancer. This study may guide the development of anticancer drugs that block the cell cycle and induce ferroptosis.

## Data Availability

The transcriptome data are deposited to NCBI Sequence Read Archive and available at https://submit.ncbi.nlm.nih.gov/subs/sra/SUB12216378/overview (BioProject ID: PRJNA895115). The mass spectrometry data for proteomics and phosphoproteomics have been deposited to the ProteomeXchange Consortium (http://proteomecentral.proteomexchange.org) (30), *via* the iProX partner repository (http://proteomecentral.proteomexchange.org/cgi/GetDataset?ID=PXD037785) with the dataset identifier PXD037785.

## Supplemental data

This article contains supplemental data.

## Ethical Approval and Consent to Participate

The animal experiments were approved by the Medical Animal Care & Welfare Committee of Jining Medical University (Approval No. JNMC-2021-DW-003).

## Conflict of interest

The authors declare no known competing interests.
