# Supplementary material for: Proteomics, Transcriptomics, and Phosphoproteomics Reveal the Mechanism of Talaroconvolutin-A Suppressing Bladder Cancer via Blocking Cell Cycle and Triggering Ferroptosis
Source: Mol Cell Proteomics. 2023 Oct 21;22(12):100672. doi: 10.1016/j.mcpro.2023.100672 (PMC10696259; doi:10.1016/j.mcpro.2023.100672)
Supplement: Supplementary Materials [file mmc1.docx]

EDC·HCl (20 mg, 0.10 mmol), biotin (12 mg, 0.047 mmol), HOBt (11 mg, 0.082 mmol), and Et_3_N (16 mg, 0.16 mmol) were dissolved in anhydrous DMF (1.5 mL) under a nitrogen atmosphere. After 10 min, Tala A (19 mg, 0.039 mmol) was added and the reaction mixture was stirred at room temperature for 24 h. Then 5 mL water was added into the mixture and extracted with ethyl acetate (3×6 mL). The combined organic solvent was washed with saturated brine, dried over anhydrous Na_2_SO_4_ and concentrated in vacuo. Purification by column chromatography (CH_2_Cl_2_/MeOH = 12:1) to provide **Biotin-TalaA** (10 mg) as a yellow soil. Yield: 36%. [α]^20^_D_ -46° (c 1.0, MeOH). ^1^H NMR (600 MHz, CDCl_3_) δ 9.46 (1H, brs), 7.58 (1H, s), 7.53 (2H, d, *J* = 8.4 Hz), 7.17 (2H, d, *J* = 8.4 Hz), 6.51 (1H, brs), 6.41 (1H, brs), 6.26 (1H, s), 5.37 (1H, s), 4.71 (1H, d, *J* = 9.6 Hz), 4.49 (1H, brs), 4.28 (1H, brs), 3.89 (1H, dd, *J* = 12.6, 7.8 Hz), 3.18-3.16 (2H, m), 2.90 (1H, d, *J* = 10.2 Hz), 2.76 (1H, d, *J* = 10.2 Hz), 2.60-2.57 (2H, m), 2.15-2.10 (1H, m), 1.85-1.68 (9H, m), 1.52-1.43 (2H, m), 1.49 (3H, s), 1.45 (3H, s), 1.24-1.20 (1H, m), 1.08-1.04 (1H, m), 0.99-0.95 (3H, m), 0.93 (3H, s), 0.85 (3H, d, *J* = 6.0 Hz), 0.75 (3H, t, *J* = 7.2 Hz), 0.68 (3H, d, *J* = 6.6 Hz). ^13^C NMR (151 MHz, CDCl_3_) δ 196.18, 172.05, 170.40, 164.49, 151.26, 144.74, 136.56, 136.44, 134.96, 132.64, 131.92, 130.97, 130.13, 129.72, 122.73, 118.84, 62.16, 60.32, 55.76, 50.91, 49.73, 48.66, 40.86, 40.14, 35.93, 35.29, 34.15, 34.02, 30.45, 28.44, 27.59, 24.83, 24.48, 22.96, 22.40, 22.37, 20.82, 20.75, 14.33, 12.20. HREIMS m/z [M]^+^ 714.3934 (calcd for C_42_H_56_N_3_O_5_S, 714.3941).


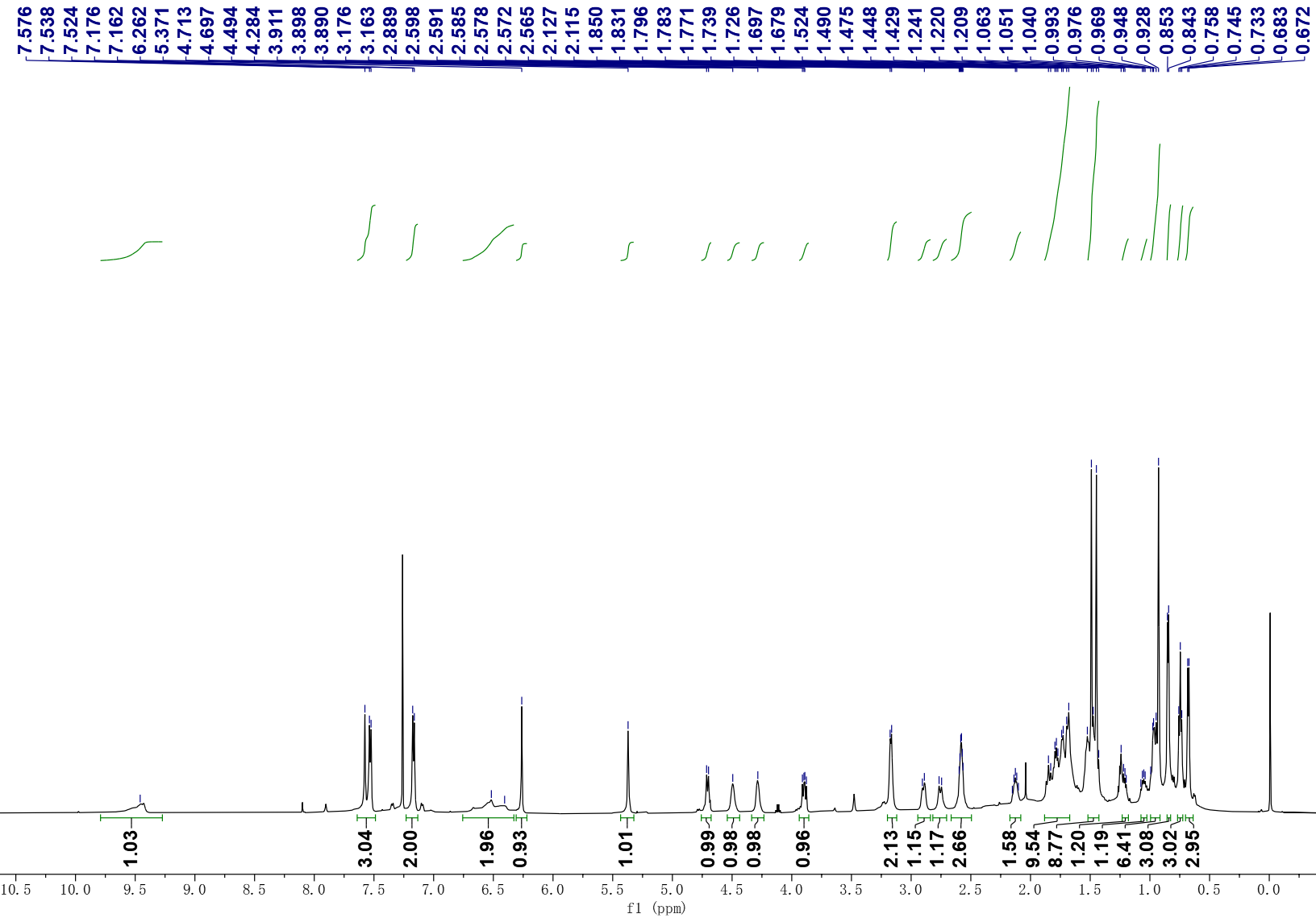


^1^H NMR spectrum (600 MHz, CDCl3) of Biotin-TalaA


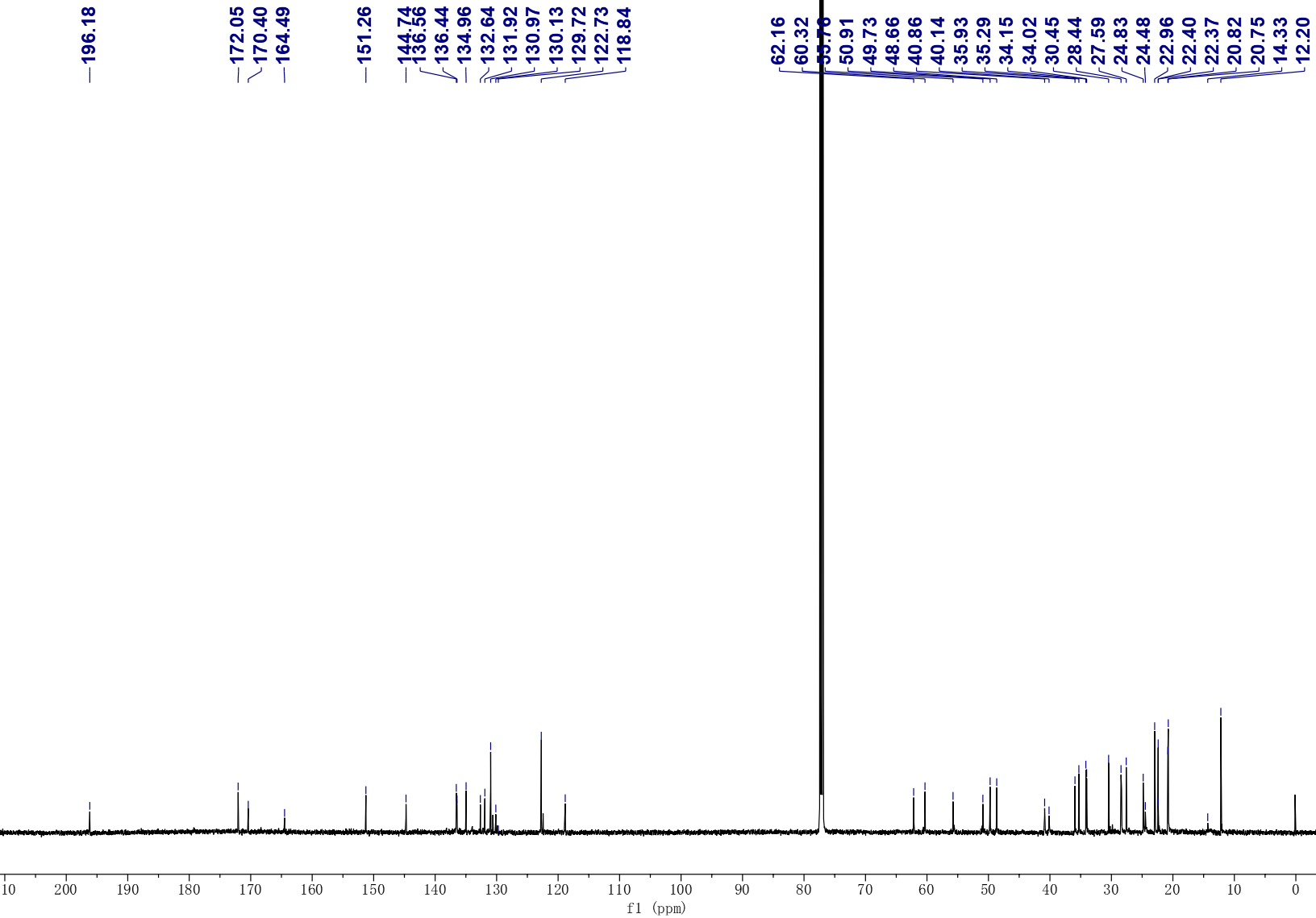


^13^C NMR spectrum (151 MHz, CDCl_3_) of Biotin-TalaA


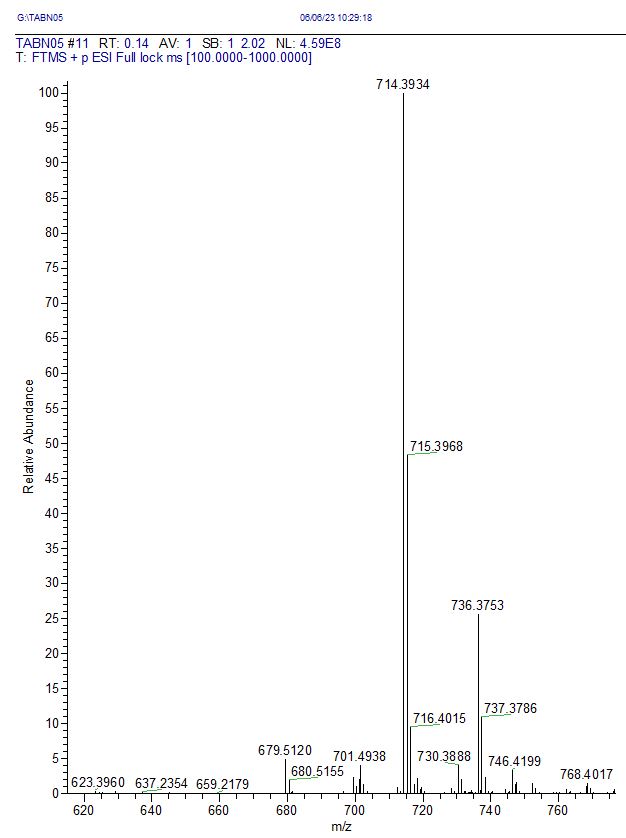


MS spectrum of Biotin-TalaA


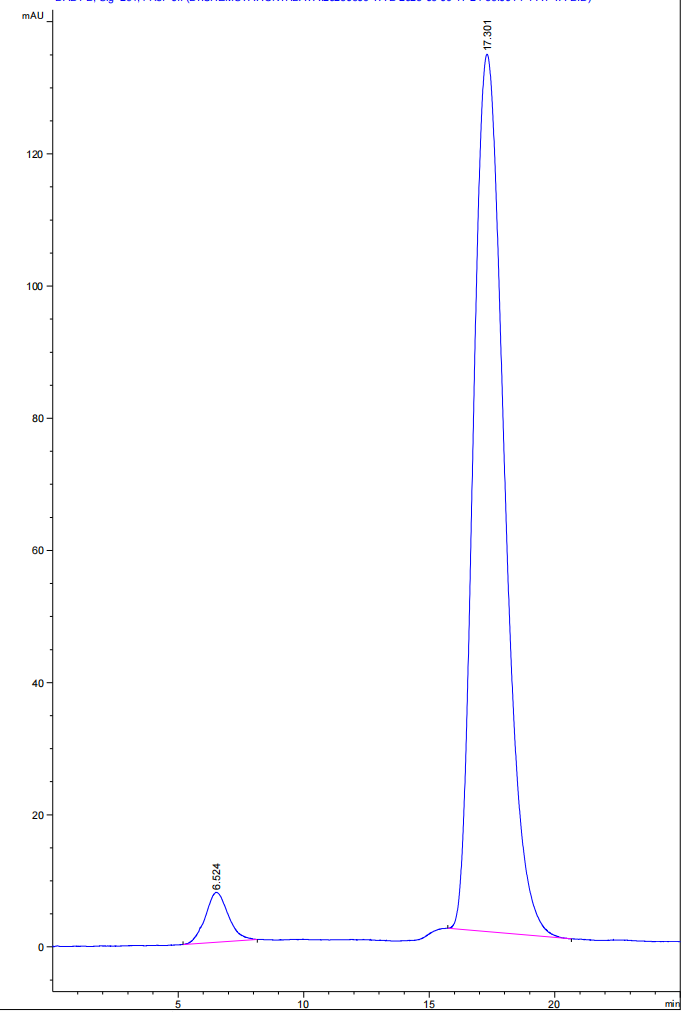


Biotin-TalaA

The purity of Biotin-TalaA was tested by HPLC (agilent-1260).


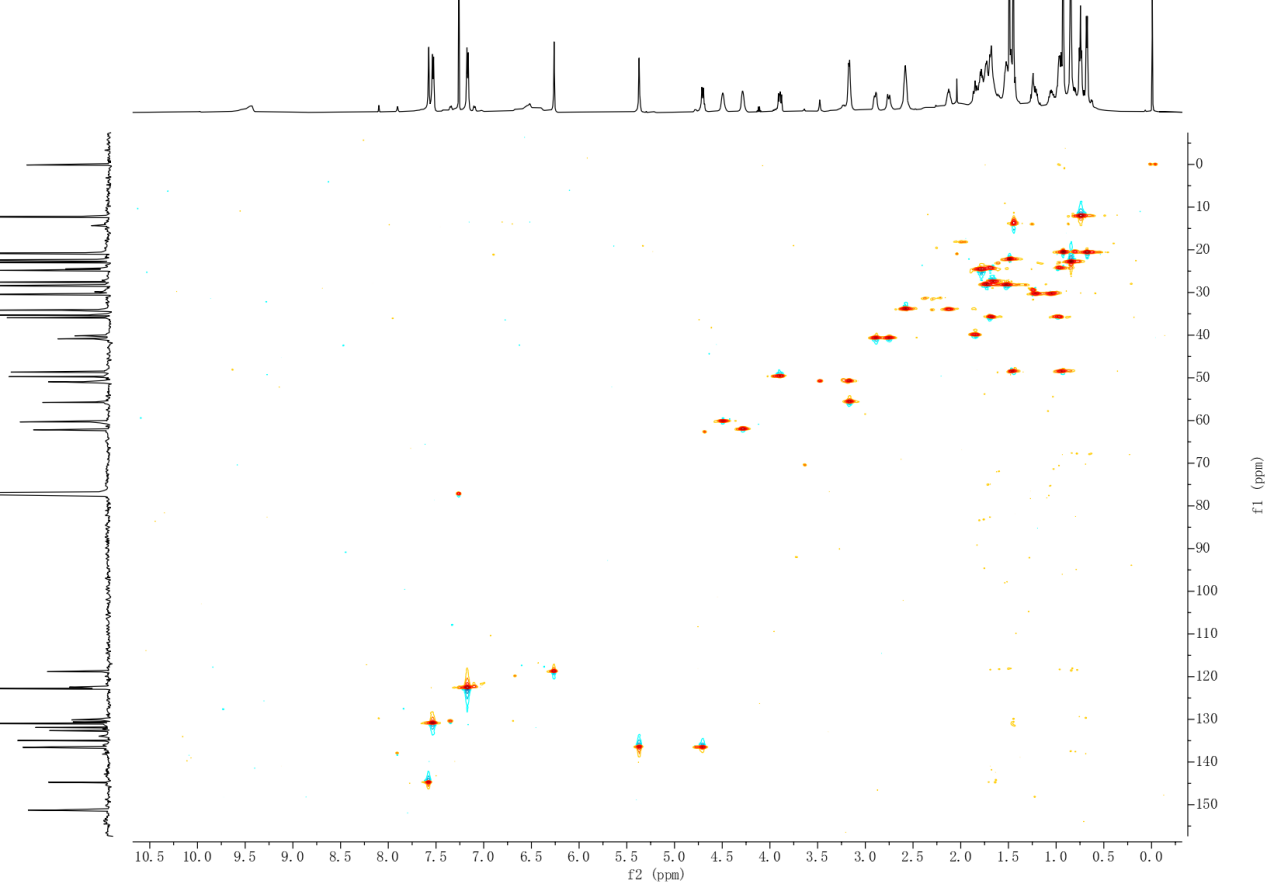


HSQC spectrum of Biotin-TalaA
